# Functional effect of Saffron supplementation and risk genotypes in early age-related macular degeneration: a preliminary report

**DOI:** 10.1186/1479-5876-11-228

**Published:** 2013-09-25

**Authors:** Dario Marangoni, Benedetto Falsini, Marco Piccardi, Lucia Ambrosio, Angelo Maria Minnella, Maria Cristina Savastano, Silvia Bisti, Rita Maccarone, Antonello Fadda, Enrica Mello, Paola Concolino, Ettore Capoluongo

**Affiliations:** 1 Dipartimento di Scienze Otorinolaringoiatriche e Oftalmologiche, Universita' Cattolica del Sacro Cuore, Lgo F. Vito 1, 00168 Roma, Italy; 2 Dipartimento di Scienze Oftalmologiche, Universita' degli Studi di Napoli Federico II, 580131 Napoli, Italy; 3 Dipartimento di Scienze Cliniche ed Applicate Biotecnologiche, DISCAB, Universita' degli Studi dell'Aquila, 67100 L' Aquila, Italy; 4 Laboratorio di Ingegneria Biomedica, Istituto Superiore di Sanità, Rome, Italy; 5 Istituto di Biochimica Clinica, Universita' Cattolica del Sacro Cuore, Lgo F. Vito 1, 00168 Roma, Italy

**Keywords:** Age-related macular degeneration, Saffron, Gene polymorphism, Electroretinography

## Abstract

**Background:**

To determine whether the functional effects of oral supplementation with Saffron, a natural compound that proved to be neuroprotective in early age-related macular degeneration, are influenced by complement factor H (CFH) and age-related maculopathy susceptibility 2 (ARMS2) risk genotypes.

**Methods:**

Thirty-three early AMD patients, screened for CFH (rs1061170) and ARMS2 (rs10490924) polymorphisms and receiving Saffron oral supplementation (20 mg/day) over an average period of treatment of 11 months (range, 6–12), were longitudinally evaluated by clinical examination and focal electroretinogram (fERG)-derived macular (18°) flicker sensitivity estimate. fERG amplitude and macular sensitivity, the reciprocal value of the estimated fERG amplitude threshold, were the main outcome measures.

**Results:**

After three months of supplementation, mean fERG amplitude and fERG sensitivity improved significantly when compared to baseline values (p < 0.01). These changes were stable throughout the follow-up period. No significant differences in clinical and fERG improvements were observed across different CFH or ARMS2 genotypes.

**Conclusions:**

The present results indicate that the functional effect of Saffron supplementation in individual AMD patients is not related to the major risk genotypes of disease.

## 

Age-related macular degeneration (AMD) is a retinal neurodegenerative disease characterized in its early stage by large soft drusen and hypo-hyperpigmentation of the retinal pigment epithelium (RPE). Late AMD, the potentially blinding stage of disease, includes geographic atrophy of the RPE (“dry” age-related macular degeneration), or subretinal neovascular membranes (“wet” age-related macular degeneration) [1].

AMD is generally considered as a multifactorial disease, whose development and progression are the results of a complex interaction between genetic and environmental risk factors. Both oxidative stress [2] and chronic inflammation [3] seem to play a significant role in the pathogenesis of AMD [4]. Risk factors as smoking [5], aging [6] or a low antioxidant intake [7] are all associated with increased levels in the outer retina of reactive oxygen species (ROS) [8] or lipid peroxidation products as malondialdehyde (MDA), potentially detrimental for photoreceptors integrity [9]. Many recent results (reviewed in Swaroop et al., 2009) [10] support the hypothesis that an uncontrolled activation of the complement system is responsible for the development of a retinal chronic inflammatory response, even in the early stages of the disease [11-14].

Several susceptibility genes have been linked to the development or progression of AMD [15-18]. The Y402H polymorphism (rs1061170) in the complement factor H (CFH) gene [19-21], and the A69S polymorphism (rs10490924) in the Age-Related Maculopathy Susceptibility 2 (LOC387715/ARMS2) [22,23] have been shown to be the strongest genetic factors to confer AMD susceptibility. CFH is the major inhibitor of the alternative complement pathway [24] and it has been suggested that a reduction in its function, leading to inadequate control of complement driven inflammation, could be responsible for the development of a retinal chronic inflammatory response, even in the early stages of AMD. Further evidences about the link between CFH and AMD derive from a recent study by Weissman et al. [25] indicating that CFH could act as a protective factor against retinal oxidative stress. In addition, the functional impact of rs1061170 CFH polymorphism on the pathophysiology of cone-mediated retinal function in AMD has been recently shown [26].

The function of ARMS2 protein remains partially unknown. Its involvement in mitochondrial function [27] and the correlation between ARMS2 polymorphisms and smoking [28,29] suggest that the gene product is implicated in oxidative stress defence.

While in the early stages of AMD visual acuity can be well preserved or only minimally reduced, functional abnormalities involving photoreceptors and/or postreceptoral neurons can be well documented by electrophysiological or psychophysical methods [30-36]. Recently, a randomized clinical trial [37], has demonstrated that dietary supplementation with Saffron is able to improve significantly the focal electroretinogram (fERG) estimated retinal flicker sensitivity in patients with early AMD, suggesting a neuroprotective effect of Saffron on dysfunctional fERG generators, namely photoreceptors, and/or bipolar cells. Further clinical evidence derives from a longitudinal study, in which a daily oral Saffron administration proved to be effective in ameliorating the macular function of early AMD patients, over a follow-up period of one year [38]. Previous experimental studies [39,40] demonstrated that Saffron may protect photoreceptors from retinal stress, preserving both morphology and function through its antioxidant and anti-inflammatory properties and probably acting as a regulator of programmed cell death.

Changes in the functional properties of proteins as CFH or ARMS2, implicated in the control of retinal inflammation/oxidative mechanisms, could influence or limit the functional response to a neuroprotective agent as Saffron. In order to test this hypothesis, we conducted a longitudinal study in early AMD patients to determine whether the effect of Saffron supplementation, evaluated by means of fERG, could be influenced by the two major AMD risk polymorphisms in CHF and ARMS2 genes.

## Methods

### Patients

Thirty-three consecutive patients (mean age, 68.4 years; range, 51–85; 15 men and 18 women) with a diagnosis of bilateral early AMD were recruited over an interval of 8 months from the outpatient service of the institution. Each patient underwent standard general and ophthalmic examinations. Clinical diagnosis of early AMD was established by direct and indirect ophthalmoscopy, as well as retinal biomicroscopy, when any of the following primary lesions in the macular area (i.e., the area within an eccentricity of approximately 2 disc diameters from the fovea) was identified: soft distinct or indistinct drusen; areas of hyperpigmentation associated with drusen; or areas of hypopigmentation of the RPE associated with drusen, without any visibility of choroidal vessels. All patients met the following inclusion criteria: best corrected visual acuity of 0.5 or better in the study eye, central fixation (assessed by direct ophthalmoscopy), normal color vision with Farnsworth D-15 testing, no signs of other retinal or optic nerve disease and clear optical media. Eight patients had moderate systemic hypertension. No other systemic diseases were present. None of the patients was taking medications (e.g., chloroquine) that are known to affect macular function or to interfere with carotenoid absorption. AMD lesions of the study eyes were graded on stereoscopic fundus photographs, as previously described [33]. A macular grading scale, based on the international classification and grading system [1] was used by a single grader who evaluated the photographs while masked to subject characteristics and fERG results. The presence of basic AMD lesions was noted within each of the nine subfields delimited by a scoring grid. Fluorescein angiography and macular optical coherence tomography (Cirrus spectral domain, Zeiss) assessment were also performed in all study eyes at the time of the diagnosis, to confirm the presence of early AMD lesions, to exclude geographic atrophy or RPE detachment, and to determine at baseline the average retinal thickness in the macular region. According to the results of grading, intermediate AMD was diagnosed in all eyes, [41] with one or more drusen (≥63 μm) and/or focal hypo-hyperpigmentation within the macular region. The average number of drusen was 9 (range, 4–22). Focal RPE abnormalities extending for at least 10% of one of the middle subfield areas in the macular region were present in 9 of 30 patients. The research protocols adhered to the tenets of the Declaration of Helsinki. The study was approved by the Ethics Committee/Institutional Review Board of the Catholic University. Written, informed consent was obtained from each study participant after the purpose and procedures of the study were fully explained.

### Treatment and testing schedule

Patients underwent clinical examination and a Focal ERG (fERG)-derived macular (18°) flicker sensitivity estimate [37] every three months over an average period of 11 months (range, 6–12) of treatment (saffron 20 mg/day) and follow-up. fERG amplitude and sensitivity, derived from the estimated response amplitude thresholds, were the main outcome measures.

Clinical and demographic data of the patients are summarized in Table 1. In all patients, a clinical examination, including visual acuity testing with a calibrated standard Snellen chart, fundus examination by direct and indirect ophthalmoscopy, and fERG testing, was performed at study entry (baseline) and every 90 days of treatment. Clinical and fERG examinations were conducted on the same day, with ophthalmoscopy always performed after fERG recordings. During the entire period of supplementation, no other systemic pharmacologic treatments were given. In all cases, compliance was judged to be satisfactory, since none of the treated subjects refrained, for any reason, from taking the daily dose of supplement during the treatment period. No adverse side effects were reported.

**Table 1:** Demographic, Genetic and Clinical Findings at baseline in Patients with early AMD

**Pt.#, gender, age**	**CFH**	**ARMS2**	**Visual acuity**	**Follow-up duration**	Fundus*	**fERG**^§^**(n. of responses at B,V3, V6, V9, V12)**
***1, F, 52***	HT	WT	0.8	6	Soft confluent drusen; central subfield	5(B), 5(V3), 5(V6)
***2, M, 63***	HT	WT	0.8	12	Soft confluent drusen and hypopigm.; central and middle subfield	4(B), 5(V3), 5(V6), 4(V9), 5(V12)
***3, M, 70***	HT	WT	0.7	12	Soft drusen; central subfield	4(B), 4(V3), 4(V6), 5(V9), 5(V12)
***4, M, 73***	HT	WT	0.7	12	Soft drusen; central and middle subfield	1(B), 5(V3), 6(V6), 6(V9), 6(V12)
***5, M, 70***	HO	WT	0.7	12	Soft confluent drusen; central subfield	5(B), 5(V3), 5(V6), 5(V9), 5(V12)
***6, M, 73***	HT	HT	1.0	12	Soft drusen; central subfield	4(B), 5(V3), 5(V6), 6(V9), 6(V12)
***7, F, 65***	HT	WT	0.9	6	Soft drusen; central subfield	4(B), 5(V3), 6(V6)
***8, M, 55***	HT	HO	1.0	12	Soft drusen and hyperpigm.; central subfield	5(B), 5(V3), 6(V6), 6(V9), 6(V12)
***9, M, 79***	HT	WT	0.5	12	Soft drusen and hyperpigm.; middle subfield	1(B), 1(V3), 4(V6), 6(V9), 6(V12)
***10, F, 77***	WT	WT	0.8	12	Soft drusen; middle subfield	4(B), 5(V3), 5(V6), 5(V9), 5(V12)
***11, M,75***	WT	HT	0.7	12	Soft drusen; middle subfield	4(B), 5(V3), 5(V6), 6(V9), 6(V12)
***12, F, 70***	HT	HT	0.7	12	Soft drusen and hyperpigm.; middle subfield	2(B), 2(V3), 1(V6), 3(V9), 3(V12)
***13, F, 81***	HT	WT	0.5	6	Soft drusen; middle subfield	4(B), 4(V3), 4(V6)
***14, F, 54***	HO	HT	0.6	12	Soft confluent drusen; middle subfield	6(B), 6(V3), 6(V6), 5(V9), 6(V12)
***15, F, 64***	HT	HO	1.0	12	Soft drusen and hypopigm.; middle subfield	2(B), 6(V3), 6(V6), 5(V9), 6(V12)
***16, M,58***	HT	WT	1.0	6	Soft drusen; middle subfield	1(B), 5(V3), 5(V6)
***17, M,73***	HT	HT	1.0	12	Soft drusen and hyperpigm.; middle subfield	4(B), 4(V3), 5(V6), 5(V9), 5(V12)
***18, M,70***	HT	WT	1.0	12	Soft drusen and hyperpigm.; central subfield	4(B), 5(V3), 5(V6), 4(V9), 4(V12)
***19, F, 71***	HO	WT	0.6	12	Soft confluent drusen and hypopigm.; central subfield	6(B), 6(V3), 5(V6), 5(V9), 6(V12)
***20, M,61***	HO	HO	0.5	12	Soft confluent drusen and hypopigm.; middle subfield	2(B), 6(V3), 5(V6), 6(V9), 6(V12)
***21, F, 62***	WT	WT	0.6	12	Soft drusen; middle subfield	6(B), 6(V3), 6(V6), 6(V9), 6(V12)
***22, F, 71***	HO	WT	1.0	12	Soft drusen; central and middle subfield	4(B), 5(V3), 5(V6), 6(V9), 5(V12)
***23, M,68***	HT	HO	0.8	12	Soft confluent drusen; central and middle subfield	2(B), 4(V3), 4(V6), 6(V9), 6(V12)
***24, M,71***	HT	HO	1.0	12	Soft confluent drusen; middle subfield	5(B), 6(V3), 6(V6), 6(V9), 5(V12)
***25, F, 81***	HT	HT	0.8	6	Soft drusen; middle subfield	4(B), 6(V3), 6(V6)
***26, F, 52***	HT	HT	0.6	12	Soft drusen; middle subfield	6(B), 6(V3), 6(V6), 6(V9), 6(V12)
***27, F, 73***	HO	WT	1.0	12	Soft drusen and hyperpigm.; middle subfield	3(B), 5(V3), 5(V6), 5(V9), 5(V12)
***28, F, 68***	HO	WT	0.6	12	Soft confluent drusen; central subfield	6(B), 5(V3), 5(V6), 5(V9), 5(V12)
***29, F,51***	WT	WT	0.5	12	Soft drusen; middle subfield	5(B), 6(V3), 6(V6), 6(V9), 6(V12)
***30, F, 84***	HT	HT	0.5	6	Soft drusen and hyperpigm.; central subfield	3(B), 3(V3), 4(V6)
***31, F, 63***	WT	WT	0.7	12	Soft drusen; central and middle subfield	5(B), 6(V3), 6(V6), 6(V9), 5(V12)
***32, M,85***	WT	WT	0.8	12	Soft drusen; central and middle subfield	5(B), 5(V3), 5(V6), 5(V9), 5(V12)
***33, F,74***	HT	WT	0.9	6	Soft drusen and hyperpigm.; middle subfield	5(B), 6(V3), 6(V6)

Follow-up duration (months). *Macular appearance with reference to drusen type, confluence, and location; RPE abnormality type and main location. ^§^Number of FERG responses that were above noise level (i.e., S/N ratio ≥ 3) at the different modulation depths of the recording protocol; (6) = S/N ratio ≥ 3 at all modulation depths, (5) = S/N ratio < 3 at the two lowest modulation depths, (4) = S/N ratio < 3 at the three lowest modulation depths, etc., B: baseline V3, V6, V9, V12: months of supplementation.

### Electrophysiological methods

fERG testing was performed according to a previously published technique [33]. Briefly, ERGs were elicited by the LED-generated sinusoidal luminance modulation of a circular uniform field (diameter, 18°; mean luminance, 80 cd/m^2^; dominant wavelength, 630 nm), presented at the frequency of 41 Hz on the rear of a Ganzfeld bowl, illuminated at the same mean luminance as the stimulus. This technique was developed according to the indications of published clinical studies, in which the fERG response to sinusoidal flicker stimulation was used to test retinal flicker sensitivity in comparison to psychophysical flicker sensitivity in normal and pathologic conditions [42,43]. In the general recording protocol, a series of fERG responses was collected at different modulation depths, quantified by the Michelson luminance contrast formula: 100% × (Lmax - Lmin)/(Lmax + Lmin), where Lmax and Lmin are maximum and minimum luminance, respectively, between 16.5% and 93.8% in 0.1- to 0.3-log-unit steps (16.5%, 33.1%, 44.8%, 63.6%, 77.2%, and 93.8%). In some patients, the signal-to-noise ratio (S/N) at the modulation of 93.5% was not large enough to allow recording of the whole response family. In those cases, response collection was limited to the highest or the two highest modulation depths. In Table 1, the number of responses that were significantly different from the noise level, collected at every visit, is reported for each patient.

fERGs were recorded monocularly by means of Ag-AgCl superficial cup electrodes taped over the skin of the lower eyelid. A similar electrode, placed over the eyelid of the contralateral, patched eye, was used as the reference (interocular ERG), [44]. fERG signals were amplified, band-pass filtered between 1 and 250 Hz (-6 dB/octave), sampled with 12-bit resolution, (2-kHz sampling rate), and averaged. A total of 1600 events (in eight blocks of 200 events each) were averaged for each stimulus condition. The sweep duration was kept equal to the stimulus period. Single sweeps exceeding the threshold voltage (25 μV) were rejected, to minimize noise coming from blinking or eye movements. A discrete Fourier analysis was performed off-line to isolate the fERG fundamental harmonic and estimate its amplitude (in μV) and phase (in degrees). Component amplitude and phase were also calculated separately for partial blocks (200-event packets) of the total average, from which the standard error of amplitude and phase estimates were derived to test response reliability. Averaging and Fourier analysis were also performed on signals sampled asynchronously at 1.1 times the temporal frequency of the stimulus, to give an estimate of the background noise at the fundamental component. An additional noise estimate at the fundamental harmonic was obtained by recording responses to a blank, unmodulated field kept at the same mean luminance as the stimulus. In all records, the noise amplitudes recorded with both methods were ≤0.053 μV.

In all subjects, the fERG testing protocol was started after a 20-minute period of preadaptation to the stimulus mean illuminance. Pupils were pharmacologically (tropicamide 1%) dilated to 8 to 9 mm. Subjects fixated (from a distance of 30 cm) on the center of the stimulation field with the aid of a small (15 minutes of arc) fixation mark. A fERG response was first collected at the maximum modulation depth (93.5%) included in the protocol and was evaluated with respect to reliability and S/N ratio. In all patients, the responses at 93.5% modulation satisfied the following criteria: standard deviation estimates of <20% (variation coefficient) and 15° for the amplitude and phase, respectively, and an S/N ratio ≥4. In AMD patients having a response S/N ≥8, fERG signals were also acquired in sequence for six values of modulation depth between 16.5% and 93.5%, presented in an increasing order. For each stimulus modulation depth, fERG responses were accepted only if their S/N ratio was ≥2. As described elsewhere [33], fERG log amplitudes were plotted for each patient as a function of log modulation depth. The resulting function slope was determined by linear regression. From the same regression line, fERG threshold was estimated from the value of log modulation depth yielding a criterion amplitude, corresponding to an S/N ratio of 3 [33].

## Genetic analysis

### Samples and DNA extraction

All patients signed an informed consent approving the use of respective specimens for the general/anonymous research protocol, in keeping with the Declaration of Helsinki. Genomic DNA was extracted from blood using High Pure PCR Template Preparation Kits (Roche Diagnostic) and quantified by the NanoPhotometer™ (Implen, Australia).

### PCR conditions and HRM acquisition

Primers were designed to amplify small fragments (81 bp and 58 bp respectively) surrounding the polymorphisms c.1277 T>C of *CFH* (rs1061170) and c.205 G>T of *ARMS2* (rs10490924): CFH-forward (CFHf) 5′-TTCCTTATTTGGAAAATGGATATAA-3′ and CFH-reverse (CFHr) 5′-GATGGCAGGCAACGTCTAT-3′; ARMS2forward (ARMSf) 5′-TGTCTTTATCACACTCCATGATCC-3′ and ARMS2reverse (ARMSr) 5′-GGTAAGCAGAGCTCAGTGTGG-3′.

PCR amplification for CFH single nucleotide polymorphism rs1061170 and ARMS2 rs10490924 was performed in a 96-well plate in the LightCycler® 480 Real-Time PCR System, as already reported in detail [45].

Genomic DNA (~ 90 ng total) was added to 10 μL of reaction master mix consisting of 1× LightCycler® 480 High Resolution Melting Master containing the proprietary ds-DNA saturating binding dye (Roche Diagnostics, Germany), in a final reaction volume of 20 μL. PCR reaction contained 3.0 mM MgCl2 and 0.15 μM of forward and reverse primers.

The PCR program started with an initial denaturation of 10 min at 95°C, continued with 50 cycles of 10 s at 95°C, 15 s at T_a_ 62/52°C and 10 s at 72°C. To allow the enrichment of the correct product over any nonspecific product we applied a touchdown PCR protocol covering a range of annealing temperature from 62 to52°C. The annealing temperature is decreased by 0.5°C every cycle. The touchdown temperature is then used for the remaining number of cycles.

The amplification step is followed by a final denaturation and reannealing step by heating to 95°C for 1min and cooling down to 40°C for 1 min. This pre-hold temperature ensures that all PCR products have re-associated and encourages heteroduplex formation.

For HRM, the plate was heated from 65°C to 95°C performing 25 acquisitions for 1°C.

### HRM analysis

Melting curve analysis was performed using the Light- Cycler® 480 Gene Scanning software version 1.5 (Roche Diagnostics, Germany).

The normalization settings were: 69.6 – 70.6°C for the pre-melting normalization and 76–77°C for the post-melting normalization for CFH, and 73 – 74°C for the pre-melting normalization and 80.6–81.6°C for the post-melting normalization for ARMS2 [45].

The melting temperature shift was performed automatically by the software using a default adjustment value of 5% for all analyses. The sensitivity level was set at 0.3, which gave sufficiently consistent and robust results. At each analytical session, unknown samples were matched with three standard samples: homozygous for the risk allele C, heterozygous, and homozygous for the allele T previously genotyped for rs1061170 and rs10490924 by sequence analysis and by comparison with the CFH and ARMS2 genomic sequence (GenBank NG_007259 and NG_011725 respectively).

Using a curve shape-matching algorithm, samples were automatically clustered into groups and the melting curve and difference plots were inspected. Significant differences in the fluorescence in all subsets indicated different genotypes.

### Statistical analysis

From each patient included in the study, one eye, typically the eye with the best visual acuity, was selected and designated as the study eye. The data from the study eyes were included in the statistical analysis. Main outcome variables were fERG amplitude, fERG function slope, and threshold. fERG amplitude data underwent logarithmic transformation to better approximate normal distribution. fERG slope and threshold are reported as log10 values. In all statistical analyses, standard error and 95% confidence interval (CI) of the means were used for between-group comparisons.

In the absence of any previous data on the effect of AMD risk polymorphisms on fERG amplitude, slope and threshold after Saffron supplementation, an accurate estimate of the statistical power of the present study could not be calculated “a priori”.

Electrophysiological results were analyzed by multivariate analysis of variance (MANOVA) for repeated measures (MANOVA). Dependent variables in the MANOVA design were fERG log amplitude, fERG threshold and slope. Stimulus modulation depth, follow-up time and genotype (wild type, heterozygous and homozygous) were the independent variables. fERG log thresholds and slopes, estimated from the corresponding functions, were individually determined by considering only responses with an S/N ≥ 3.

In all the analyses, results with a p < 0.05 were considered statistically significant.

## Results

Table 1 reports the results regarding the genotype distribution for either CFH and ARMS2 polymorphisms in each patient. We found 27 out of 33 patients (82%) carrying the “C” allele for the CFH gene (20 in the hetero and 7 in the homozygous state, respectively), while only 13 out of 33 patients (39%) carried the “T” allele in the ARMS2 gene (8 in the hetero and 5 in the homozygous state, respectively). Visual acuity did not significantly differ between patients carrying or not the single variants, alone or in combination. Similarly, age and funduscopic features were closely matched between the different genetic subgroups. There was no significant variation across groups in the number or size of drusen as well as in the extent of RPE abnormalities.

### CHF

**Figure 1:**
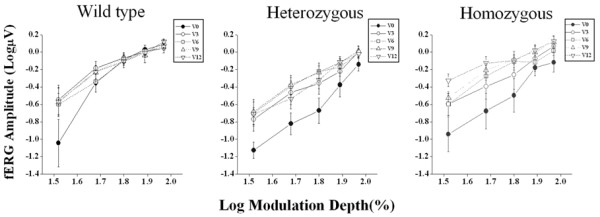
**fERG modulation function in patients carriers of the CFH (rs1061170) polymorphism.** Mean fERG amplitudes (± SE) as a function of modulation depth, at baseline (V0) and after three (V3), six (V6), nine (V9) and twelve (V12) months of Saffron treatment, are represented separately for wild type (n = 6), heterozygous (n = 20) and homozygous (n = 7) patients for the CFH (rs1061170) polymorphism.

The results of fERG modulation functions recorded in wild type, heterozygotes and homozygotes for the CFH (rs1061170) polymorphism, at baseline and every three months after starting Saffron supplementation are reported in Figure 1. Responses at the lowest modulation value (16.5%) are not shown because in most patients they were recordable only after saffron supplementation. There was an overall increase in fERG amplitude after the first three months of supplementation followed by stabilization over the subsequent follow-up period. MANOVA showed a significant effect of Saffron treatment (F (4, 19) = 3.89; p < 0.01) and modulation depth (F (4, 19) = 68.4; p < 0.01) on amplitude. No significant interaction effect between genotype and Saffron treatment was found comparing the three different genotype groups.

**Figure 2:**
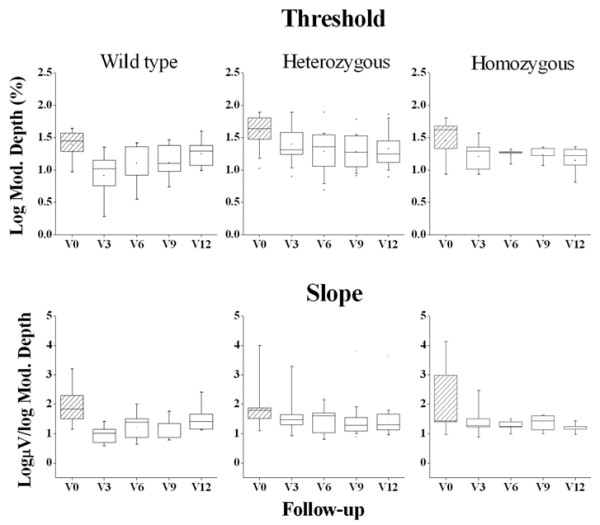
**fERG threshold and slope in patients carriers of the CFH (rs1061170) polymorphism.** Box-whiskers plots of fERG threshold and slope values at baseline (V0) and after three (V3), six (V6), nine (V9) and twelve (V12) months of Saffron treatment, in wild type (n = 6), heterozygous (n = 20) and homozygous (n = 7) patients, for the CFH (rs1061170) polymorphism. In each diagram the symbol is the mean, the box indicates the median and interquartile range and bars indicate the 95 percentiles.

Figure 2 shows box plots of the distribution of mean fERG thresholds and slopes (± interquartile and 95 percentile ranges) recorded in wild type, hetero and homozygous patients for the CFH (rs1061170) polymorphism, at baseline and every three months after Saffron supplementation. As shown in the Figure, the mean threshold decreased (and consequently the reciprocal sensitivity value increased) in each group of patients after the first three months of Saffron supplementation. MANOVA showed a significant effect of Saffron treatment on fERG threshold (F (4, 20) = 6.2; p < 0.01) but no differences across genotype groups. Mean fERG slope did not change significantly throughout the follow-up period.

### ARMS2

**Figure 3:**
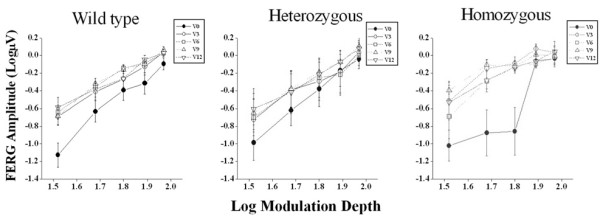
**fERG modulation function in patients carriers of the ARMS2 (rs10490924) polymorphism.** Mean FERG amplitude (± SE) as a function of the corresponding values of modulation depth, at baseline (V0) and after three (V3), six (V6), nine (V9) and twelve (V12) months of Saffron treatment, are represented separately for wild type (n = 20), heterozygous (n = 8) and homozygous (n = 5) patients for the ARMS2 (rs10490924) polymorphism.

The results of fERG modulation functions recorded in wild type, heterozygotes and homozygotes for the ARMS2 (rs10490924) polymorphism, at baseline and every three months after starting Saffron supplementation are reported in Figure 3. MANOVA showed a significant effect of Saffron treatment (F (4, 18) = 6.46; p < 0.01) and modulation depth (F (4, 18) = 62.1; p < 0.01) on amplitude, and a significant interaction effect between genotypes carrying “T” variant and modulation depth (F (8, 36) = 2.29; p < 0.05). No significant interaction effect between genotype and Saffron treatment was found comparing the three different genotype groups.

**Figure 4:**
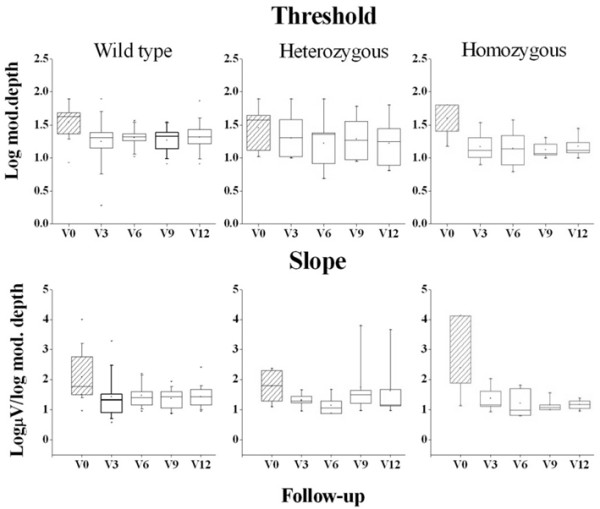
**fERG threshold and slope in patients carriers of the ARMS2 (rs10490924) polymorphism.** Box-whiskers plots of fERG threshold and slope values at baseline (V0) and after three (V3), six (V6), nine (V9) and twelve (V12) months of Saffron treatment, in wild type (n = 20), heterozygous (n = 8) and homozygous (n = 5) patients, for the ARMS2 (rs10490924) polymorphism. In each diagram the symbol is the mean, the box indicates the median and interquartile range and bars indicate the 95 percentiles.

Figure 4 shows box plots of the distribution of mean fERG thresholds and slopes (± interquartile and 95 percentile ranges) recorded in wild type, hetero and homozygous patients for the ARMS2 (rs10490924) polymorphism, at baseline and every three months after Saffron supplementation. Mean threshold decreased (consequently the reciprocal sensitivity value increased) in each genotype group after the first three months of Saffron supplementation. MANOVA showed a significant effect of Saffron treatment on fERG threshold (F (4, 19) = 6.9; p < 0.01). No significant differences in terms of threshold changes were revealed across groups. Mean fERG slope did not change significantly throughout the follow-up period.

## Discussion

In the present study we evaluated whether the functional effects of Saffron supplementation on retinal function of early AMD patients could be influenced by CFH or ARMS2 risk genotypes. The improvement of macular function after Saffron supplementation, as evidenced by an increase in retinal flicker sensitivity, did not differ significantly between patients who were hetero/homozygous or those who were not carriers of CHF or ARMS2 polymorphisms, indicating that the individual’s response to Saffron treatment is not dependent on CHF or ARMS2 genotypes. Moreover, Saffron treatment proved to exert a long term efficacy in all genotypes. The improvement of macular function observed after three months of Saffron supplementation, remained stable throughout the 12 months follow-up in all patients, regardless of genotype.

The Y402H CHF polymorphism is a major genetic risk factor associated to AMD [46]. CFH represents the major fluid phase inhibitor of the alternative complement pathway [47], and therefore has an anti-inflammatory activity. Any change in CFH regulation can have a profound effect on the alternative pathway regulation [47] leading to an uncontrolled activation of the alternative complement pathway and to the development of a complement driven inflammation, potentially detrimental for the retinal tissue [48]. New evidences about the existing link between CHF and AMD derive from a recent study by Weismann et al. [25], who have demonstrated that CHF can directly protect the retina from oxidative damage and that Y402H variant is able to increase retinal susceptibility to an inflammatory damage induced by oxidative stress. As a consequence of an altered CHF, fERG generators, i.e., photoreceptors and bipolar cells, would be exposed to a greater inflammatory/oxidative stress resulting more damaged and dysfunctional, as evidenced by a lower fERG amplitude and retinal sensitivity in patients who are carriers of the Y420H polymorphism [26]. This finding is further supported by a study of Feigl et al. [49] who showed that carriers of the Y402H polymorphism, even with a normal fundus and visual acuity, may have a significantly lower psychophysical flicker fusion frequency, as compared to normal/low risk genotypes, with mesopic stimulation. In this context, the beneficial functional effects of Saffron supplementation found in this study, may indicate that the early damaging effect of AMD on the outer retina, exacerbated by the CFH risk genotypes, can be significantly counteracted by this approach.

An example of how risk polymorphisms could influence the efficacy of an antioxidant supplementation in AMD patients can be found in a recent pharmacogenetic study by Klein et al. [50]. In this work the authors evaluated the influence of the Y402H CFH and the A69S ARMS2 polymorphisms on the response to treatment with Age-Related Eye Disease Study (AREDS) formulation (Zinc, β-carotene, Vitamin C and E), in reducing risk progression towards advanced stages of AMD. AREDS supplementation was associated with a greater reduction in AMD progression (68%) in patients who were not carriers of the Y402H polymorphism, as compared with those who were homozygous (11%), indicating that CFH (rs1061170) can limit significantly the benefits deriving from the AREDS formulation. Conversely, ARMS2 polymorphism did not influence AREDS individual’s response.

The mechanisms by which Saffron exerts its neuroprotective action on retinal cells are complex and still under investigation. Preclinical studies have demonstrated the antioxidant [39,51], anti-inflammatory [52-54] and antiapoptic [55] properties of this compound. In an experimental model [40] of photoreceptor light damage in albino rat retina, Saffron-treated animals showed preservation of the photoreceptor morphology and function, as well as a reduced number of apoptotic figures. There is evidence that Saffron inhibits TNF-α induced apoptosis, by modulating Bcl-2 family protein expression [55] and suppresses caspase activation [56]. Therefore, Saffron seems to exert its neuroprotective action on retinal cells acting on different levels, protecting, upstream, retinal cells from potential apoptotic stimuli thanks to its antioxidant and anti-inflammatory properties, and controlling, downstream, directly the apoptosis process, interfering with the intracellular decisional mechanisms that lead the cell to initiate the apoptotic program.

One of the major limitations of the present study is represented by the reduced sample size of the patients that were included, a factor that could account for a low statistical power. However it should be considered that is the first study that has tried to quantify in terms of retinal sensitivity changes, the pharmacogenetic interaction between the AMD associated risk polymorphisms and a treatment with an oral supplementation. It is therefore reasonable to consider the present work a preliminary pharmacogenetic report, and even if larger studies are needed to confirm its results, it can provide, to our opinion, useful indications for the design of further pharmacogenomic association studies in AMD patients.

## Conclusion

In conclusion, the results of this study indicate that Saffron long term efficacy in improving macular function is rather independent by the two major CFH and ARMS2 risk polymorphisms related to AMD. This could have direct implications for the clinical management of early AMD patients, in whom appropriate neuroprotective strategies may be critical in preventing the development of the blinding stage of disease.

## Abbreviations

AMD: Age-related macular degeneration; AREDS: Age-related eye disease study; ARMS2: Age-related maculopathy susceptibility 2; CFH: Complement factor H; fERG: Focal electroretinogram; MANOVA: Multivariate analysis of variance; MDA: Malondialdehyde; ROS: Reactive oxygen species; RPE: Retinal pigment epithelium.

## Competing interests

Prof. Bisti and Dr. Maccarone hold a non remunerative relationship with “Hortus Novus”, the company that provided the Saffron pills used in this study. None of the other authors have any actual or potential conflicts of interest related to this study.

## Authors’ contributions

DM and BF conceived of the study and drafted the manuscript. MP and LA performed the focal electroretinogram in the patients and collected the data. AMM and MCS participated in the selection and in the clinical management of the patients. SB and RM participated in the study design and coordination and helped to draft the manuscript. AF performed the statistical analysis and helped to draft the manuscript. EM ad PC carried out the genetic analysis of the patients and helped to draft the manuscript. EC conceived of the study, carried out the genetic analysis and revised critically the manuscript. All authors read and approved the final manuscript.
